# The involvement of MALAT1-ALKBH5 signaling axis into proliferation and metastasis of human papillomavirus-positive cervical cancer

**DOI:** 10.1080/15384047.2023.2249174

**Published:** 2023-08-28

**Authors:** Suzhen Wu, Lili Liu, Huanying Xu, Qiaoling Zhu, Minhua Tan

**Affiliations:** Department of Obstetrics & Gynecology, Foshan Fosun Chancheng Hospital, Foshan, Guangdong, P.R. China

**Keywords:** MALAT1, ALKBH5, proliferation, migration, invasion, HPV-positive cervical cancer

## Abstract

Infection with high-risk human papillomavirus (HPV), for example, with types 16 and 18, is closely associated with cervical cancer development, which continues to threaten women’s health globally. Although HPV oncogenes have been recognized as the main cause of transformation of normal cervical epithelial cells, non-coding RNA could also be involved in the initiation and promotion of cervical cancer development. Metastasis-associated lung adenocarcinoma transcript 1 (MALAT1), a well-documented long non-coding RNA (lncRNA), has been previously reported to exert roles in HPV-positive cervical cancer; however, the detailed underlying mechanism has yet to be investigated. In the present study, high expression levels of MALAT1 in HPV-Positive Cervical Cancer cells were confirmed, and silencing MALAT1 resulted in decreased rates of cell proliferation, migration, and invasion, both *in vitro* and in a zebrafish xenograft tumor model. Moreover, the results obtained showed that silencing MALAT1 led to down-regulation of the N^6^-methyladenosine (m6A) demethylase ALKBH5 via regulating miR-141-3p expression, which caused a decrease in the expression levels of matrix metalloproteinase 2 (MMP2) and MMP9 expression, thereby suppressing cell migration and invasion. Taken together, the results obtained have suggested that the MALAT-ALKBH5 signaling axis may be activated in HPV-positive cervical cancer cells, which could contribute to cell proliferation and metastasis through the regulation of key genes, such as MMP2 or MMP9. The findings of the present study should both help to improve our understanding of the underlying tumorigenic mechanisms of HPV-positive cervical cancer and be of further use in the development of potential therapeutic drugs.

## Introduction

Globally, cervical cancer (CC) ranks as the second most common malignant tumor, and the second leading cause of cancer-associated deaths among women.^1^ Over the past few decades, although the treatment and diagnosis of cervical cancer have met with significant improvements, the 5-year overall survival rate for patients with metastatic cervical cancer remains very low.^2^ Advanced-stage cervical cancer has been characterized as the invasion of cancerous cells to adjacent areas and metastasis to lymph nodes and distant tissues, processes that are responsible for most cervical cancer -associated mortality.^3^ Patients infected with high-risk human papillomavirus (HR-HPV), especially types 16 and 18, account for ~ 70% of the cases of invasive cervical cancer cases.^4–6^ Therefore, gaining an improved understanding of the molecular mechanisms underlying the metastasis of HPV-positive cervical cancer would be beneficial in terms of working toward its prevention, diagnosis, and treatment.

Long non-coding RNAs (lncRNAs) are biologically active transcripts > 200 nucleotides in length that themselves lack protein-coding function, which have been documented to regulate gene expression and protein functions associated with a wide spectrum of cellular and physiological functions.^7^ Previous studies have shown that lncRNAs fulfill crucial roles in the initiation and progression of tumors, including cervical cancer. For example, the lncRNA CAR10 was shown to promote cervical cancer progression via influencing the microRNA (miRNA) miR-125b-5p and upregulating the expression of 3-phosphoinositide-dependent protein kinase 1.^8^ The lncRNA SNHG12 was shown to contribute to the development of cervical cancer predominantly through modulating the miR-125b/STAT3 axis in a variety of cervical cancer cell lines.^9^ Furthermore, Jin *et al*.^10^ demonstrated that the level of the lncRNA TCONS_00026907 was markedly higher in cervical cancer tissues compared with non-cancerous tissues, and subsequently performed *in vitro* experiments to show that silencing lncRNA TCONS_00026907 lead to inhibition of cervical cells. As one of the first lncRNAs to be identified and studied, metastasis-associated lung adenocarcinoma transcript 1 (MALAT1) is located on chromosome 11q13.1, is highly conserved among mammals, and has been shown to be aberrantly expressed in cervical cancer.^11^ Several previously published studies have suggested that MALAT1 can influence cervical cancer cell proliferation, invasion, and angiogenesis by acting as a microRNA (miRNA) precursor or miRNA sponge, thereby influencing the post-transcriptional regulation of gene expression.^12,13^ Whether there are other MALAT1-associated signaling pathways that are involved in cervical cancer, however, needs to be investigated further.

The N^6^-methyladenosine(m6A) modification is the most abundant and reversible conserved post-transcriptional modification in mammalian cells, which is regulated by a series of “writer,” “reader,” and “eraser” proteins.^14^ The modification of m^6^A is involved in the development of multiple diseases, including the occurrence and progression of a variety of tumors.^15^ As an m6A eraser protein, AlkB homolog 5 RNA demethylase (ALKBH5) directly catalyzes the removal of m6A modifications/^16^ A recent study demonstrated that silencing of ALKBH5 in glioblastoma cells led to the suppression of cell proliferation and invasiveness.^17^ Interestingly, Chen *et al*.^18^ demonstrated that ALKBH5 functions a tumor suppressor, and overexpression of ALKBH5 was shown to reduce hepatocellular carcinoma cell proliferation and invasiveness. These effects could be due to different mRNAs being targeted by ALKBH5 in different cancer cells, for example, FOMX1 in glioblastoma, LYPD1 in hepatocellular carcinoma, PD-L1 in Intrahepatic Cholangiocarcinoma, and so on.^18–20^ Furthermore, ALKBH5 has also been shown to function as a regulator of miRNA or lncRNA expression to promote tumorigenesis.^21^ However, at present, the role of ALKBH5 in cervical cancer development remains poorly understood.

The present study aimed to investigate the role of MALAT1 in HPV-positive cervical cancer cells. To meet this end, MALAT1 was found to be upregulated in HP-positive cervical cancer cells, and this upregulation contributed to the proliferation and metastasis of cervical cancer cell both *in vitro* and in a zebrafish xenograft tumor model. Interestingly, silencing of MALAT1 led to a significant downregulation of ALKBH5 expression, rather than that of other epigenetic regulators. In addition, the present study will demonstrate that ALKBH5 exerts roles in the proliferation and metastasis of HPV-positive cervical cancer cells. Mechanistically, MALAT1 upregulates ALKBH5 probably through affecting miR-141-3p expression, which consequently leads to increased expression levels of the matrix metalloproteinases (MMP2) and MMP9, eventually leading to the metastasis of HeLa and CaSki cells. Considered altogether, the findings of the present study extend our understanding of the role of MALAT1-ALKBH5 signaling axis in HPV-positive cervical cancer cell metastasis and invasion, thereby providing both novel insights into the underlying mechanism and potential new avenues for therapeutic interventions in HPV-positive cervical cancer.

## Materials and methods

### Cell cultures

C33A, HeLa, and CaSki cells were purchased from the National Collection of Authenticated Cell Cultures. C33A and HeLa cells were maintained in Gibco® Dulbecco’s modified Eagle’s medium (DMEM) (Thermo Fisher Scientific, Inc), whereas CaSki cells were cultured in Gibco® RPMI 1640 medium (Thermo Fisher Scientific, Inc.). All media were supplemented with 10% Gibco® fetal bovine serum (FBS) (Thermo Fisher Scientific, Inc) and 1% penicillin/streptomycin (Beyotime Institute of Biotechnology). Cells were kept in an incubator in a humidified atmosphere of 5% CO_2_ at 37°C.

### Plasmids and transient transfection

The pcDNA3.1 vector, pcDNA3.1-MALAT1, and miR-141-3p mimics (5’- TAACACTGTCUGGTAAAGATGG-3’) were purchased from General Biosystems (Anhui) Co., Ltd. (Chuzhou, China), and the coding sequence of ALKBH5 gene (NM_017758.4) was inserted to generate the pcDNA3.1-ALKBH5 construct. Transient transfection (2 μg of DNA) was performed using Invitrogen® Lipofectamine^TM^ 3000 reagent (Thermo Fisher Scientific, Inc) according to the manufacturer’s instructions.

### RNA interference (RNAi) assay

HeLa and CaSki cells were transfected with siNC, siMALAT1, or siALKBH5, respectively, using Invitrogen® Lipofectamine^TM^ 3000 reagent (Thermo Fisher Scientific, Inc) according to the manufacturer′s instructions. After 48 hours, cells were collected for validation of the efficiency of transfection and for subsequent experiments. All of the aforementioned siRNAs were purchased from Anhui General Biology Co., Ltd. The siRNA sequences were as follows:

siNC, 5’-UUCUCCGAACGUGUCACGU-3’; si1-MATAL1, 5’-UGCCUUUAGGAUUCUAGACA-3’; si2-MALAT1, 5’-CCAGGCUGGUUAUGACUCAG-3’; si1-ALKBH5, 5’-GCUGCAAGUUCCAGUUCAA-3’; si2-ALKBH5, 5’-UGAUACUUGCGCUUGGCCC-3’; si1-FTO, 5’-GGAAGAAGAUGGAGGGUGU-3’; si2-FTO,5’-AAGAUGAAGUGGACAUUAA-3’; si3-FTO, 5’-CGGUGGCAGUGUACAGUUA-3’; si1-METTL3,5’-CAGUGGAUCUGUUGUGAUA-3’; si2-METTL3, 5’-UGGCAUGAUUGAAAGACUA-3’; si3-METTL3, 5’-GUAUGAACGGGUAGAUGAA-3’; si-PVT1, 5’-CAGCCATCATGATGGTACT-3’.

### Cell proliferation assay

Cells were inoculated at a density of 5 × 10^3^ cells per well in 96-well plates in 6 replicate wells and incubated for 24 h, followed by the corresponding treatments. Cell Counting Kit-8 (CCK-8; 10 μl) reagent (Beyotime Institute of Biotechnology) was added to each well at 24, 48, 72, and 96 h post-transfection, followed by the incubation for a further 2 h at 37°C. Finally, the absorbance (450 nm) was measured using an ELx-800 microplate reader (BioTek Instruments, Inc.).

### Cell migration and invasion assay

For cell migration assay, cells were seeded at a density of 5 × 10^3^ cells per well in the upper chamber of 24-well polycarbonate membrane inserts with 8.0 μM pore size (Corning, Inc.). For the invasion assays, the inserts were pre-coated with Matrigel^TM^ solution (Corning, Inc.) in the upper chamber. Serum-free medium was added to the upper chamber, and the lower chamber was filled with complete medium with 20% FBS. After 24 h, following removal of the cells on the top surface of the upper chambers, the remaining cells were fixed in 4% paraformaldehyde and then stained with 1% crystal violet (MilliporeSigma) for 20 min. After washing with PBS, the transmembrane cells were photographed and counted the number of migrated cells for quantification under an I× 53 microscope (magnification 100 ×) (Olympus, Tokyo, Japan).

### RNA extraction and reverse transcription-quantitative PCR (RT-qPCR)

After the corresponding treatments, cells were collected and total RNA was extracted using an RNA isolation kit (Vazyme Biotech Co., Ltd.). Subsequently, 1 µg samples of RNA were reverse-transcribed, and qPCR reactions were performed with specific primers using regent kits from Vazyme Biotech Co., Ltd. Glyceraldehyde-3-phosphate dehydrogenase (GAPDH) or U6 was used as the internal reference gene, and the fold changes of expression were calculated using the 2^−(ΔΔCT)^ method. All primers were synthesized by General Biosystems (Anhui) Co., Ltd., and their sequences are shown in Table 1.

**Table 1.** 

	5’−3’
GAPDH-F	GGGAGCCAAAAGGGTCAT
GAPDH-R	GAGTCCTTCCACGATACCAA
MALAT1-F	AGCGGAAGAACGAATGTAAC
MALAT1-R	GAACAGAAGGAAGAGCCAAG
MMP2-F	CTGCGGTTTTCTCGAATCCATG
MMP2-R	GTCCTTACCGTCAAAGGGGTATCC
MMP9-F	GAGGCGCTCATGTACCCTATGTAC
MMP9-R	GTTCAGGGCGAGGACCATAGAG
METTL3-F	TTGTCTCCAACCTTCCGTAGT
METTL3-R	CCAGATCAGAGAGGTGGTGTAG
METTL14-F	GTTGGAACATGGATAGCCGC
METTL14-R	CAATGCTGTCGGCACTTTCA
WTAP-F	ACTGGCCTAAGAGAGTCTGAAG
WTAP-R	GTTGCTAGTCGCATTACAAGGA
ALKBH5-F	CGGCGAAGGCTACACTTACG
ALKBH5-R	CCACCAGCTTTTGGATCACCA
FTO-F	GCTGCTTATTTCGGGACCTG
FTO-R	AGCCTGGATTACCAATGAGGA
MALAT1-F	AGCGGAAGAACGAATGTAAC
MALAT1-R	GAACAGAAGGAAGAGCCAAG
PVT1-F	TTGGCACATACAGCCATCAT
PVT1-R	CAGTAAAAGGGGAACACCA
miR-141-3p-F	GTTTGGTAACACTGTCTGGTAA
miR-141-3p-R	GTGCAGGGTCCGAGGT
U6-F	GCTTCGGCAGCACATATACTAAAAT
U6-R	CGCTTCACGAATTTGCGTGTCAT

### Western blot analysis

Cells were lysed using RIPA buffer (Beyotime Institute of Biotechnology), and the protein concentration was determined by using Pierce® BCA protein assay (Thermo Fisher Scientific, Inc.) using bovine serum albumin as a standard. Equal amounts of denatured proteins (30 μg) were subjected to SDS-PAGE electrophoresis and subsequently transferred to PVDF membrane (MiliporeSigma). After blocking the membranes with 5% defatted milk for 1 h, the membrane was incubated with the primary antibodies (see below) at 4°C overnight. After washing the membrane three times, the secondary antibody (see below) was added at room temperature for 1 h. Finally, the immunoblots were viewed using ECL Developer (Beyotime Institute of Biotechnology) and photographed with a gel imager (Bio-Rad Laboratories, Inc.). ImageJ software was used to calculate the relative gray values using β-actin as loading control. The antibody information was as follows: Primary antibody: anti-ALKBH5 antibody (1:1000, Cell Signaling Technology, Inc.), anti-MMP2 antibody (1:1000, Elabscience Inc.), anti-MMP9 antibody (1:1000, BOSTER Biological Technology Co. Ltd.), and anti-GAPDH antibody (1:5000, Beyotime Institute of Biotechnology); and secondary antibody: HRP conjugated anti-rabbit or -mouse secondary antibody (1:5000, MultiSciences Biotech Co., Ltd.).

### Zebrafish in vivo study

Zebrafish were raised at 28°C in a 14h/10h light–dark cycle in a fish auto-culture system (Shanghai Haisheng Biological Experiment Equipment Co., Ltd.). Fish embryos were raised in 10% Hank’s solution. The zebrafish AB wild-type and transgenic line Tg (fli1a: EGFP) were employed in the present study. Transfected cells were collected in serum-free medium and subsequently stained with 1 μg/ml Celltracker CM-DiI solution (Shanghai Yeasen Biotechnology Co., Ltd.) for 15 min in the dark, the labeled cells at a density of 2 × 10^6^ cells/ml were then microinjected into the yolk space of 2 dpf zebrafish juveniles within 2 h. Approximately 400 cells were injected into each fish, followed by incubation at 37°C for 96 h. Successful transplantation was confirmed and photographed using fluorescence stereomicroscope (MVX10, Olympus). The average intensity of fluorescent cells in yolk (proliferation) and trunk (metastasis) of fish was quantified by Image J software. The representative pictures were taken via confocal microscope (Fluoview 3000, Olympus). Fish was euthanized by rapid chilling in ice water for 30 min according to the AVMA guidelines for the euthanasia of animals (2020 Edition). All the animal experiments were performed in accordance with the guidelines of the Provision and General Recommendation of Chinese Experimental Animals in China and were approved by the Ethics Committee of Foshan Fosun Chancheng Hospital.

### Statistical analysis

All data are presented as the mean ± SD. Statistical analysis was performed using the one-way ANOVA with Bonferroni’s test for multiple comparison and unpaired t‐test for comparison between two groups. All experiments were performed in triplicate, unless otherwise stated. *P < .05, **P < .01 and ***P < .001 were considered to indicate statistically significant differences.

## Results

### Effect of MALAT1 on the growth and metastasis of HPV-positive cervical cancer in vitro and vivo

**Figure 1.**
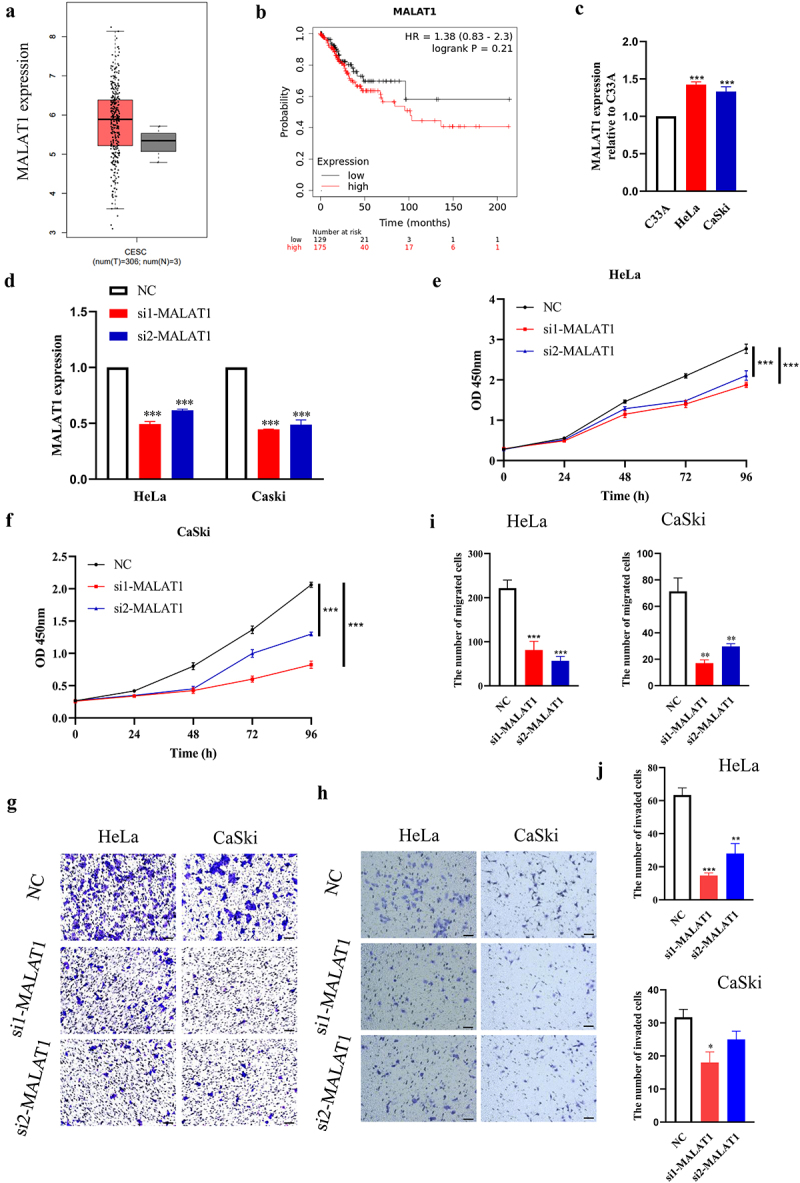
Expression and effect of MALAT1 on cervical cancer cells *in vitro*.

First, the expression level of MALAT1 in tumor and normal tissue samples was analyzed through Gene Expression Profiling Interactive Analysis (GEPIA) (http://gepia.cancer-pku.cn/), showing that higher MALAT1 was observed in cervical tumor tissue (Figure 1a). In addition, patients with higher MALAT1 seemed to have lower overall survival rates according to Kaplan–Meier analysis (https://kmplot.com/analysis/) (Figure 1b). To further verify the expression of MALAT1, the mRNA level of MALAT1 in three different cervical cancer cells, namely the C33A (HPV-negative), HeLa, and CaSki (HPV positive) cell lines, was determined. As shown in Figure 1c, compared with the C33A cell lines, the Hela and CaSki cell lines exhibited higher levels of MALAT1 expression, implying the casual association of MALAT1 with HPV infection. Using a specific siRNA targeting MALAT1 in HPV-positive cells (Figure 1d), the results obtained showed that downregulation of MALAT1 significantly suppressed the proliferation of the two HPV-positive cervical cancer cell lines  (Figure 1e). Migration and invasion assay were subsequently employed to evaluate the effects of MALAT1 on cell metastasis *in vitro*. As a result, transfection of the Hela and CaSki cells with siMALAT1 attenuated the numbers of migrated and invaded cells, respectively, through the Transwell pores compared with the numbers using negative control siRNA (Figure 1g–j). Collectively, these results demonstrated that MALAT1 may promote the proliferation and metastasis of HPV-positive cervical cancer cell *in vitro*.

(a) The expression of MALAT1 in tumor samples was analyzed. (b) The overall survival rate of patients with high and low MALAT1 expression was analyzed. (c) The expression level of MALAT1 was determined by RT-qPCR. Fold changes are shown using C33A cells as control. (d) HeLa and CaSki cells were transfected with NC and two specific siRNAs against MALAT1 (si1 and si2), and the expression of MATAL1 was measured using RT-qPCR. Quantitative data are shown. (e and f) Cells were treated the same as described in (d), and cell growth assay was performed at different time points, as indicated. (g-j) Cell migration and invasion assay were performed after transfection of siRNAs for MATAL1 in Hela and CaSki cells, respectively. The representative fields were photographed (G : migration, H: invasion) (scale bar: 100 μm), and the numbers of migrated and invaded cells were calculated. Three independent experiments were performed. NC, negative control.

**Figure 2.**
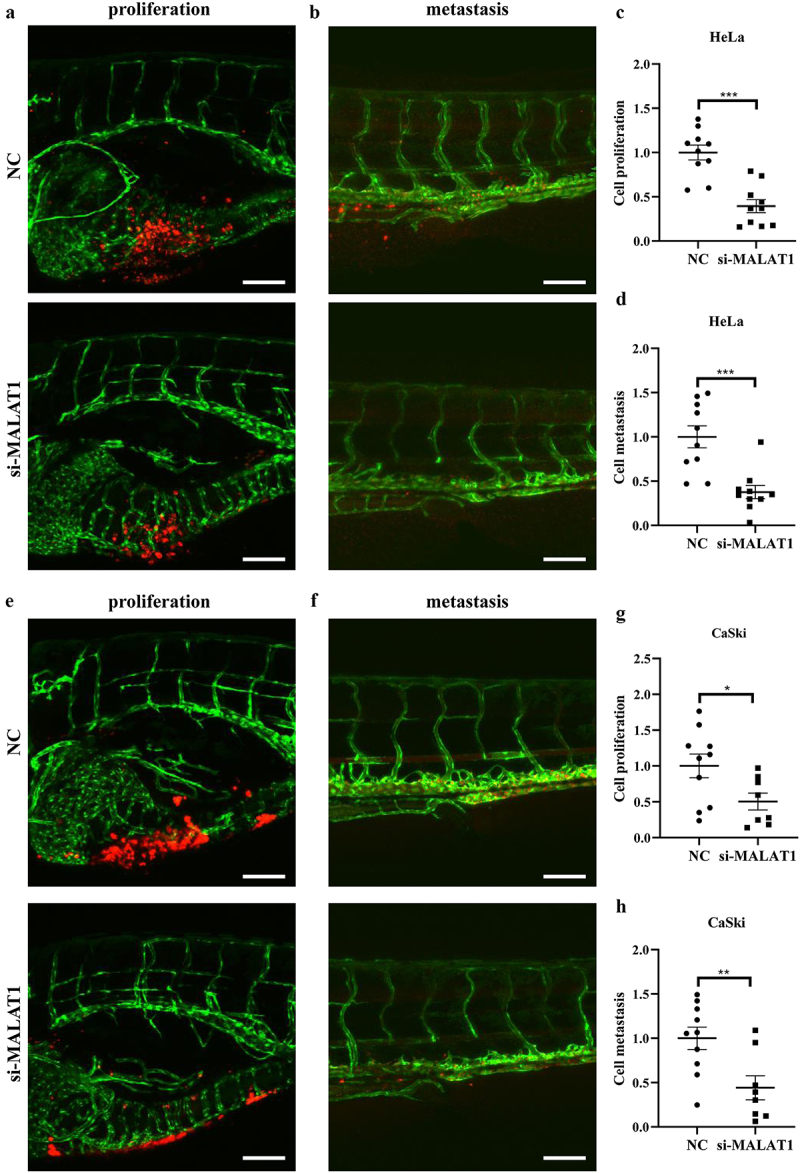
Effect of MATAL1 knockdown on cell proliferation and metastasis *in vivo*.

The zebrafish xenograft model has been developed as an ideal tool to observe tumor cell proliferation and metastasis, which has the intrinsic advantages of scale, cost, time, and multiplexing of conditions compared with 2D cultures.^22^ In this context, zebrafish larvae were chosen to investigate the effect of MALAT1 on HPV-positive cervical cancer *in vivo*. As shown in Figures 2a & 2c, knockdown of MALAT1 in Hela cells significantly decreased their proliferation rate, as viewed with red fluorescence in the zebrafish fish bellies and quantification of cell fluorescence in yolk, compared with negative control group (Fig. S2). Meanwhile, compared with Hela cells transfected with negative control siRNA, transfection of siMALAT1 disrupted the dissemination of the HeLa cells to the distant tail within the zebrafish, indicating the metastasis was attenuated (Figures 2b & 2d, Fig. S2). The same experiments were performed using the CaSki cells, and similar results (Figure 2e–h, Fig. S2) were observed. Taken together, the data suggested that downregulating MALAT1 in HPV-positive cervical cancer could suppress tumorigenesis.

HeLa and CaSki cells were transfected with NC and siRNA against MATAL1, respectively, and labeled cells were then transplanted into larval zebrafish to evaluate cell proliferation and metastasis *in vivo*, as described in the Materials and methods section. (a and b) Representative photos of zebrafish injected with HeLa cells were taken using a confocal laser scanning microscope for each treatment. To quantify the proliferation and metastasis of injected cells, the zebrafish larvae were imaged with stereomicroscope. The fluorescence of HeLa cells that (c) resided in the fish yolk or (d) trunk was quantified using Image J software, and the values were normalized against those of the NC (*n* = 10). (e-h) Representative pictures (left panel) and quantified results (right panel) are shown for CaSki cells, as above (*n* = 10) (scale bar: 100 μm). NC, negative control.

### MATAL1 regulates ALKBH5 through affecting miR-141-3p

**Figure 3.**
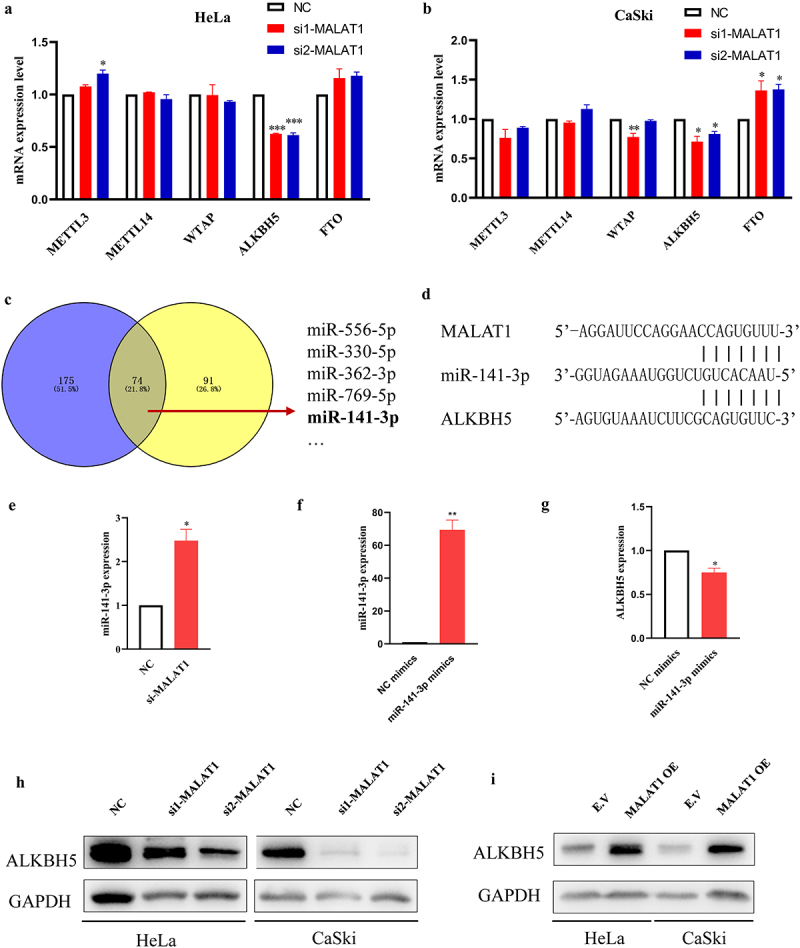
Effect of MATAL1on ALKBH5 expression in HeLa and CaSki cells.

It has been well documented that m6A modification of non-coding RNA is able to influence their expression. However, in these experiments, no expected downregulation of MALAT1 as previously reported was observed after silencing METTL3 and FTO, both of which are key players in RNA m6A modification^23,24^ (Fig. S1). Given that lncRNAs also regulated the core m6A methyltransferase complexes,^25^ we were wondering whether MALAT1 could exert a similar effect. Interestingly, the data shown in Figure 3a, b revealed that MALTAT1 was especially associated with one of m6A demethylases, ALKBH5, but not with the other genes encoding m6A methyltransferase (METTL3/METTL14/WTAP) or demethylase (FTO) in HPV-positive cervical cancer cells. LncRNAs could serve as competing endogenous RNAs (ceRNAs) that indirectly regulate gene expression by sequestering miRNA away from target mRNA. To determine whether MALAT1 regulated ALKBH5 by acting like ceRNAs, the potential target miRNAs were predicted by StarBase (https://starbase.sysu.edu.cn/). As shown in Figure 3c, there were over 70 miRNAs that could bind both MALAT1 and ALKBH5 mRNA. Among these miRNAs, miR-141-3p was selected for the following investigation because it was recently documented to directly target ALKBH5 expression and involve in prostate cancer progression (Figure 3d).^26^ As a result, silencing MALAT1 increased miR-141-3p expression, while miR-141-3p mimics downregulated the mRNA expression level of ALKBH5 (Figure 3e–g). Furthermore, knockdown of MALAT1 significantly reduced, whereas overexpression of MALAT1 upregulated, the ALKBH5 expression in both Hela and CaSki cells (Figure 3h, i, Fig. S3). Collectively, these data suggested that MATAL1 could function as an upstream regulator for ALKBH5 gene expression probably through acting as miR-141-3p sponges.

(a and b) NC and two siRNA (si1 and si2) for silencing MALAT1 were transfected into HeLa and CaSki cells. The expression of the m6A-associated genes (METTL3, METTL14, WTAP, ALKBH5, and FTO) was determined by RT-qPCR. (c) The candidate miRNAs, which might bind to MALAT1 or ALKBH5, were predicted by StarBase. (d) Sequence of miR-141-3p was matched with MALAT1 and ALKBH5 3’UTR. (e) The expression of miR-141-3p was analyzed after knockdown of MALAT1 in HeLa cells. (f) The expression of miR-141-3p was analyzed after transient transfection of miR-141-3p mimics in HeLa cells using RT-qPCR. (g) The mRNA expression level of ALKBH5 was analyzed by RT-qPCR following transfection of miR-141-3p mimics in HeLa cells. (h) The cell lysates were subjected to western blot analysis for ALKBH5 expression after silencing MALAT1 using GAPDH as loading control. (i) The MALAT1 was overexpressed followed by analysis of ALKBH5 protein expression in HeLa and CaSki cells. NC, negative control.

### Involvement of ALKH5 in growth and metastasis of HPV-positive cervical cancer cells in vitro and in vivo

**Figure 4.**
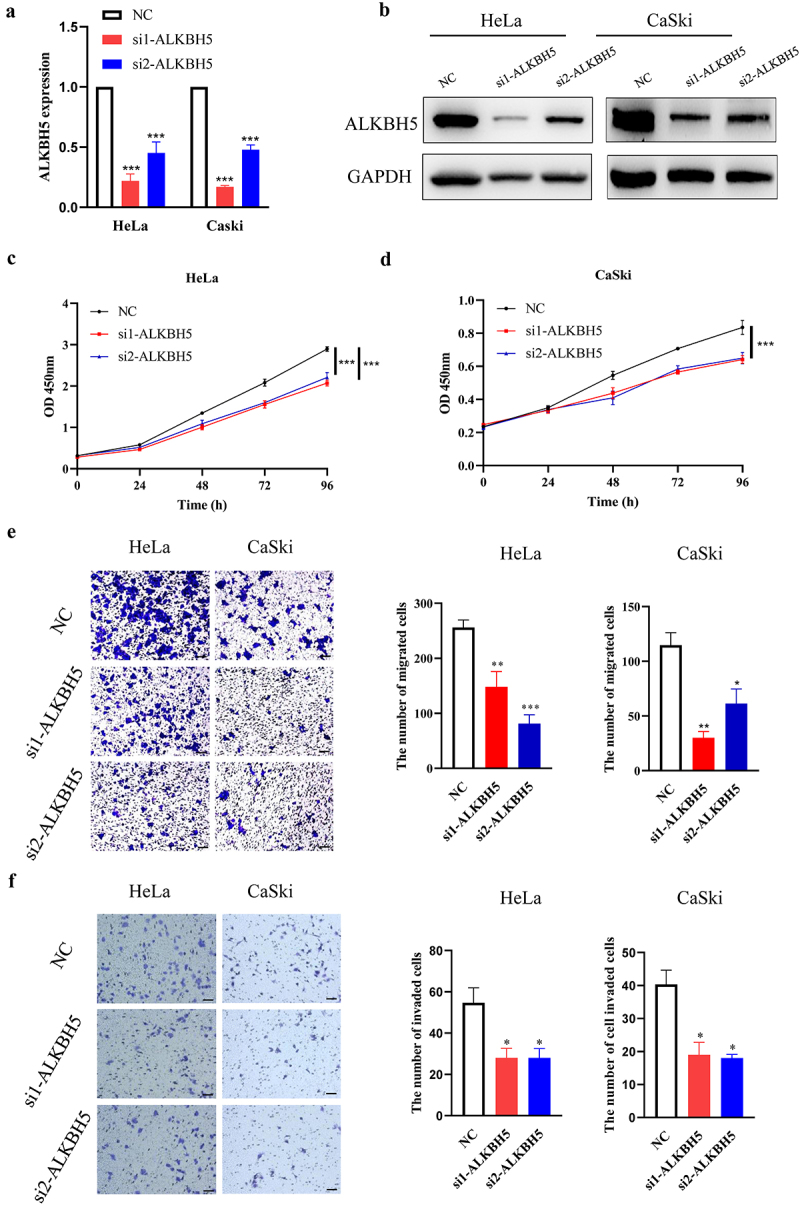
Effect of ALKBH5 on proliferation, migration and invasion in HeLa and CaSki cells.

**Figure 5.**
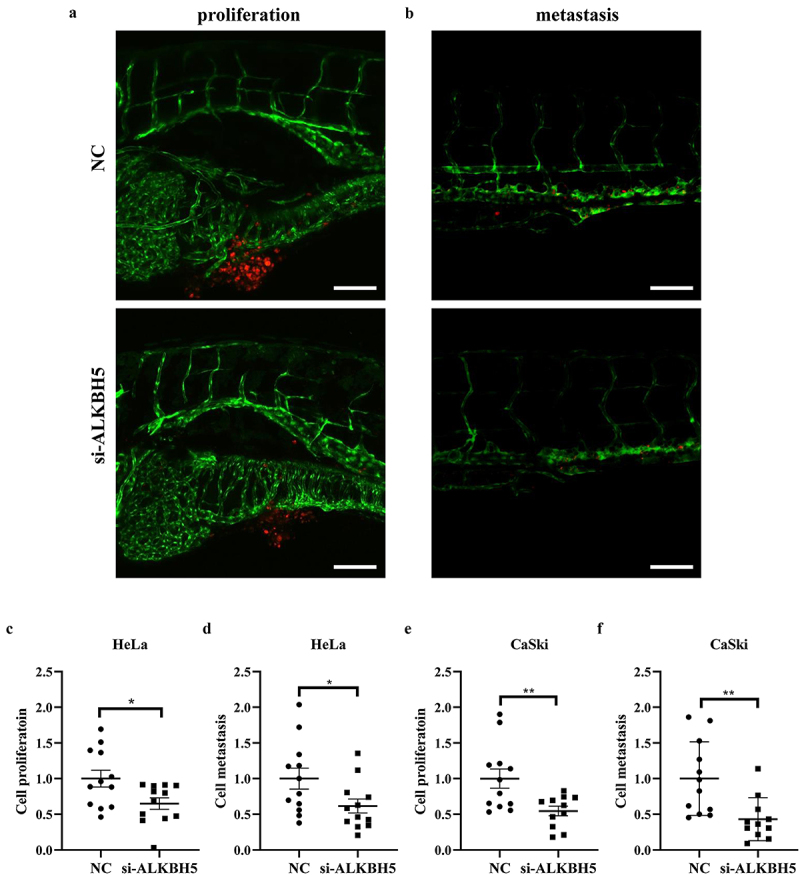
Effect of ALKBH5 on cell proliferation and metastasis *in vivo*.

Since the previous experiments performed in this study have shown that MATAL1 was able to upregulate ALKBH5 expression, it was reasonable to surmise that ALKBH5 could contribute to the development of HPV-positive cervical cancer. To clarify the role of ALKBH5 in cervical cancer, ALKBH5 was downregulated using specific siRNAs in both Hela and CaSki cells (Figure 4a, b). As shown in Figure 4c, d, silencing ALKBH5 in Hela and CaSki cells inhibited cell proliferation as evaluated by cell fluorescence in fish yolk (Fig. S4). On the other hand, the significant decrease in number of migrated and invaded cells that were observed as a consequence of downregulating ALKBH5 expression suggested that the major function of ALKBH5 could be in enhancing metastasis in cervical cancer (Figure 4e, f, Fig. S4). Moreover, the zebrafish xenograft experiments showed that HPV-positive cervical cancer cells in which ALKBH5 was silenced were propagated and spread to a lesser extent compared with the negative control group (Figure 5a–f, Fig. S4). Collectively, these results demonstrated that ALKBH5, as a downstream target of MATAL1, was also able to promote HPV-positive cervical cancer development.

HeLa and CaSki cells were transfected with NC and two siRNAs (si1 and si2) against the ALKBH5 gene. (a and b) The cells were harvested, and total RNA and protein were isolated. Total RNA was reverse-transcribed into cDNA for measuring ALKBH5 mRNA expression using RT-qPCR, and the lysates were subjected to western blot analysis using a specific antibody against ALKBH5 protein with GAPDH as the control. (c and d) Cell proliferation assay was performed at the indicated times for both HeLa and CaSki cells. (e) Cell migration and (f) invasion assays were performed. Representative fields were photographed (scale bar: 100 μm) (left panel), and the number of migrated and invaded cells was subsequently calculated (right panel). NC, negative control.

HeLa and CaSki cells were transfected with NC and two siRNAs (si1 and si2) against the ALKBH5 gene, and labeled cells were transplanted into larval zebrafish to observe cell growth and metastasis. (a and b) Representative photos for proliferation (yolk) and metastasis (trunk) of HeLa cells in a zebrafish model taken by confocal laser scanning microscope are shown (scale bar: 100 μm). (c-f) The fluorescence of HeLa and CaSki cells in different sites of zebrafish was visualized by stereomicroscope and quantified using Image J software and normalized by negative control (*n* = 12). NC, negative control.

### Effect of the MALAT1/ALKBH5 signaling axis on MMP2 and MMP9

**Figure 6.**
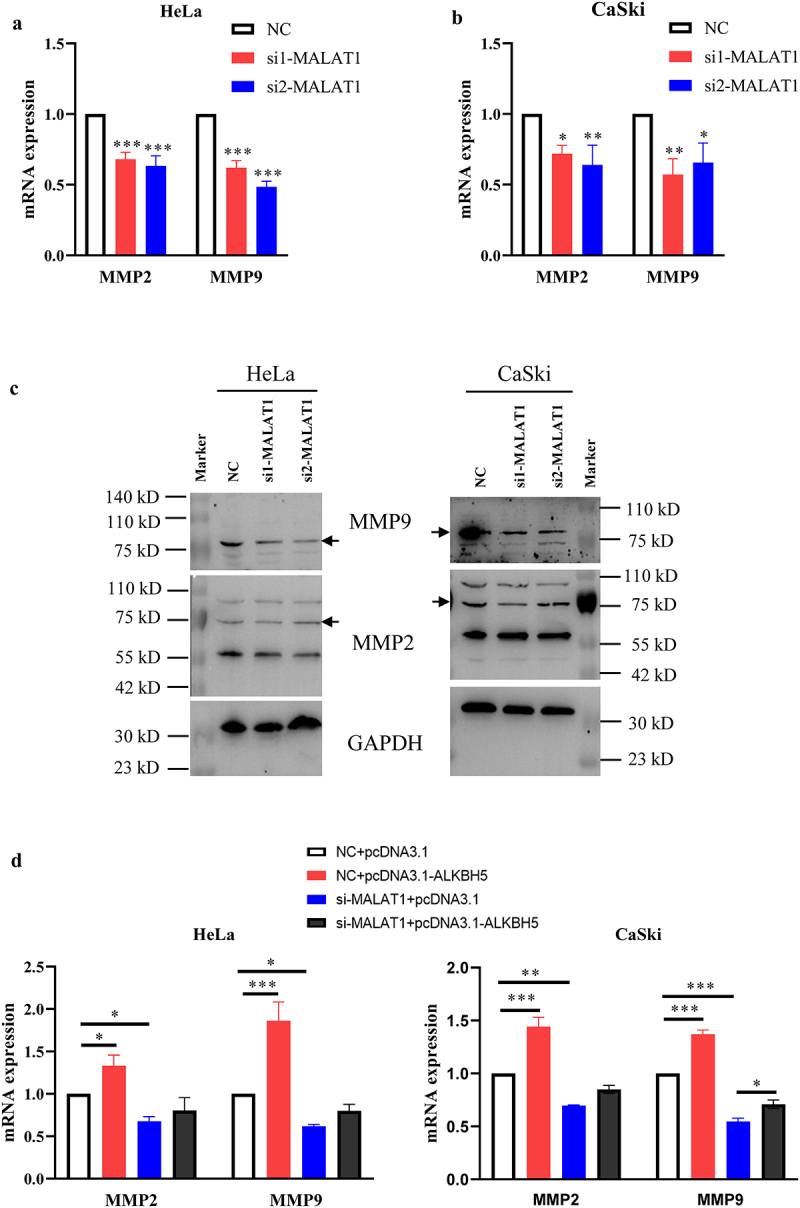
Effect of the MALAT1-ALKBH5 axis on MMP2 and MMP9 expression.

As the data described above have shown, MALAT1 could affect an increase in ALKBH5 expression, and both of them involved in the growth and metastasis of HPV-positive cervical cancer cells. Given the key roles mediated by MMP2 and MMP9 in tumor metastasis, their association with MALAT1 and ALKBH5 was subsequently investigated. As a result, knockdown of MALAT1 significantly reduced the expression levels of MMP2 and MMP9 at both mRNA and protein level in Hela and CaSki cells, respectively (Figure 6a–c). By contrast, overexpression of MALAT1 resulted in the increase in mRNA expression level of MMP2 and MMP9 (Fig. S5A and B). Moreover, overexpression of ALKBH5 led to a partial restoration of the MMP2 and MMP9 expression levels which were downregulated in the presence of siMALAT1 in both cell lines (Figure 6d and S5C and D). In addition, silencing ALKBH5 also downregulated the mRNA expression level of MMP2/9 independent of mRNA stability regulation (Fig. S6A-D). Interestingly, PVT1, one of lncRNA, was influenced by ALKBH5 silencing and associated with MMP2/9 expression in cervical cancer cells (Fig. S6E and F). Considered together, these data suggested that MALAT1 can increase ALKBH5 expression, which further upregulates the expression levels of MMP2 and MMP9 possibly through affecting the endogenous RNA crosstalk network.

(a and b) HeLa and CaSki cells were, respectively, transfected with NC and two siRNAs against MALAT1 or ALKBH5. The expression levels of MMP2 and MMP9 was determined by RT-qPCR. (c) The cell lysates were subjected to western blot analysis of MMP2 and MMP9 after knockdown of MALAT1 in HeLa and CaSki cells using GAPDH as loading control. (d) Both HeLa and CaSki cells were transfected with NC and siRNAs against MALAT1 with co-transfection of the ALKBH5 expression vector (pcDNA3.1-ALKBH5), with empty vector (pcDNA3.1) serving as the control. Cell RNA was isolated and subsequently reverse transcribed into cDNA, which were subjected to RT-qPCR for determination of MMP2 and MMP9 expression using specific primers. NC, negative control.

### Effect of the MALAT1/ALKBH5 axis on migration and invasion in vitro

**Figure 7.**
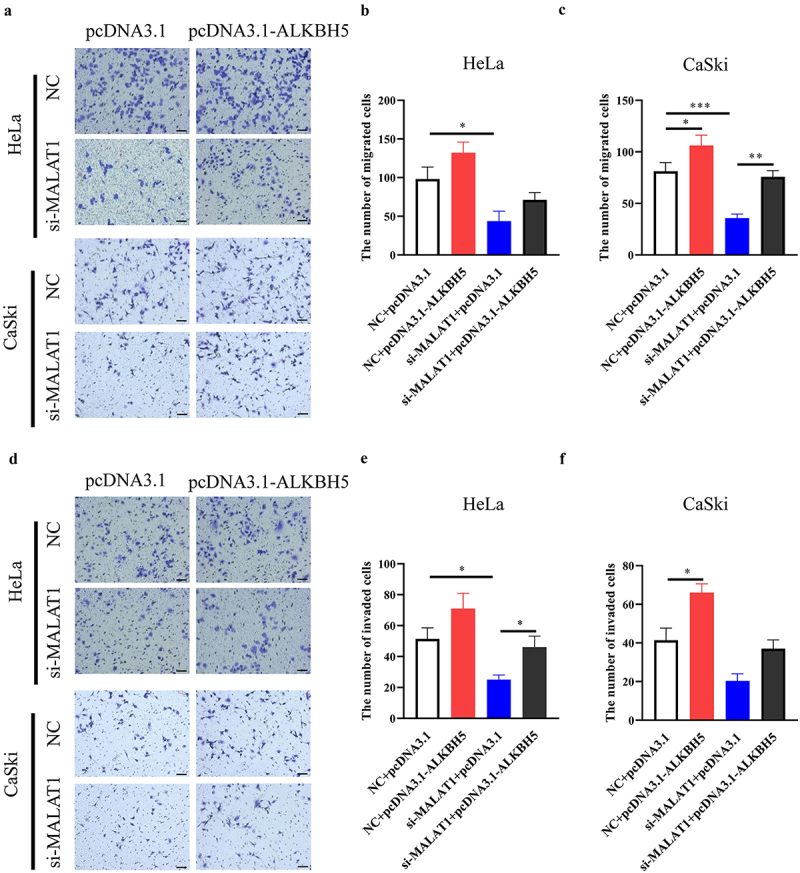
Effect of ALKBH5 on suppression of cell migration and invasion by silencing MALAT1.

To further verify that ALKBH5 is a downstream effector of MALAT1, migration and invasion assays in HPV-positive cervical cancer cells in the presence of si-MALAT1 were performed with or without overexpression of ALKBH5. Overexpression of ALKBH5 was verified at mRNA and protein level in both HeLa and CaSki cells (Fig. S5C and D). Then, as shown in Figure 7a–f, ALKBH5 overexpression alone could enhance cell migration and invasion, which further confirmed the role of ALKBH5 in the metastasis of cervical cancer. As was observed previously, siMALAT1 alone could suppress the migration and invasion of Hela and CaSki cells, whereas overexpression of ALKBH5 partially counteracted these effects (Figure 7a–f). These data suggested that MALAT1 is able to promote migration and invasion, partly via upregulating ALKBH5 expression in HPV-positive cervical cancer cells.

Both HeLa and CaSki cells were transiently transfected with siMALAT1 and ALKBH5 expression vector alone or combination. (a-c) Cell migration assay was performed, and the representative fields were photographed (scale bar: 100 μm). The numbers of migrated cells were also counted based on three independent experiments. (d-f) Cell invasion assay was also performed, and the representative photos and the numbers of invaded cells are shown as described above.

## Discussion

Cervical cancer is ranked as the second leading cause of cancer mortality of females in China; 90% of HPV-associated cancers are cervical cancer.^27,28^ It is generally accepted that the metastasis and recurrence of cervical cancer are the most challenging problems confronting the success of therapeutic measures, and these are also the most important factors affecting the prognosis of patients with cervical cancer.^29^ Further investigation of these problems is necessary, since multiple mechanisms may be involved in HPV-positive cervical cancer metastasis.

MALAT1, a classical lncRNA, is able to target genes either by directly controlling their expression or through modulating miRNAs indirectly and participating in tumor migration, invasion, and proliferation in various types of cancer.^30^ A previous study documented that the association of MALAT1 with an increased incidence of lung cancer brain metastasis.^31^ In addition, overexpression of MALAT1 has been shown to confer drug resistance in ovarian cancer and contribute to epithelial–mesenchymal transition in esophageal cancer.^32^ MALAT1 was shown to inhibit the radio-sensitivity of HPV-positive cervical cancer and to promote the chemo-resistance of cervical cancer^33^. In the present study, it was shown that the downregulation of MALAT1 suppressed the cell growth and metastasis of HPV-positive cervical cancer cells *in vitro* and in a zebrafish tumor model, findings that were in line with the observation described above. Although m6A methyltransferases or demethylases such as METTL3 and FTO have been identified as having roles in the regulation of MALAT1 expression,^23,34^ in the present study silencing of the expression of these two genes did not cause any alterations in MALAT1 expression. Whether HPV oncogenes may result in higher level of MALAT1 remains unknown.

Interestingly, the present study did demonstrate that MALAT1 could regulate ALKBH5, one of m6A demethylases, which could be involved in miR-141-3p expression. It has been reported that ALKBH5-mediated m6A RNA demethylation affects mRNA export.^35^ More importantly, ALKBH5 is involved in the initiation and development of different types of cancers, and its expression also varies with different cancer types. ALKBH5 is aberrantly expressed in glioblastoma, and silencing of ALKBH5 was shown to inhibit the proliferation of glioblastoma.^19^ ALKBH5 upregulation has been positively correlated with the development of breast and leukemia cancer.^36^ In addition, ALKBH5 was downregulated in hepatocellular carcinoma, which was shown to be a predictor of poorer survival.^18^ The levels of ALKBH5 were also decreased in bladder cancer tissues compared with adjacent normal tissues.^37^ In the present study, the effect of ALKBH5 on HPV-positive cervical cancer cells was first evaluated, demonstrating that decreased ALKBH5 reduced the growth and metastasis of HPV-positive cervical cancer cells, whereas ALKBH5 overexpression enhanced cell migration and invasion but did not significantly alter cell growth. Very recent study showed that miR-141-3p directly targeted ALKBH5 and promoted prostate cancer progression.^26^ In addition, MALAT1 has been documented to regulate miR-141-3p expression in other cells.^38,39^ In agreement with previous reports, we confirmed MALAT1 affected miR-141-3p in cervical cancer cells, which might result in the alteration of ALKBH5. However, the complicated signaling networks should be further investigated to rule out the involvement of other pathways.

Previous studies have shown that ALKBH5 could inhibit tumor growth and metastasis in lung cancer via regulating Yes-associated protein (YAP), P53, and transforming growth factor (TGFβ)/Smad signaling pathways.^40–42^ However, other studies reported that ALKBH5 may promote the proliferation and metastasis of different types of cancer, including lung cancer via FOXM1 mRNA demethylation, which improves its stability.^19,43,44^ Collectively, these studies have suggested that ALKBH5 may modulate growth and metastasis through multiple mechanisms, depending on cellular context. Herein, the results obtained showed that MALAT1 was associated with MMP2 and MMP9 expression, and that its effects may be mediated via ALKBH5 expression. Due to the association of ALKBH5 with the TGFβ/Smad signaling pathway, it is not surprising that MMPs can be regulated by ALKBH5. However, the results obtained from this study suggest that PVT1 could be involved in MMP2 and MMP9 regulation by ALKBH5 in cervical cancer cells. Considering the complicated crosstalk networks of different types of endogenous RNA in cells, it remains to be carefully clarified the underlying mechanisms. Nevertheless, overexpression of ALKBH5 only partially restored the levels of MMP2 and MMP9, as well as the numbers of migrating and invading cells, all of which were suppressed by silencing MALAT1, suggesting that there could be other possible targets affected by MALAT1. It is also probable that the activation of the MALAT1-ALKBH5 axis is able to affect various signaling pathways besides those influenced by MMP2 and MMP9. Given the powerful roles of ALKBH5 in m6A modification, it is worthwhile to identify the potential targets of ALKBH5 at the whole transcriptome level in HPV-positive cervical cancer using MeRIP-seq assay, which would be further investigated in our future study. In addition, whether the MALAT1-ALKBH5 axis is involved in HPV-negative cervical cancer, and the manner in which way HPV infection is associated with the activation of this pathway, are largely unanswered questions that required further investigation.

Taken together, the results obtained in the present study have shown the involvement of the MALAT1-ALKBH5 signaling axis in proliferation, migration, and invasion of HeLa and CaSki cells, which may function through upregulating the gene expression of factors associated with metastasis, such as MMP2 and MMP9. These results contribute toward a deeper understanding of the underlying mechanism of HPV-positive cervical cancer development and may be beneficial in terms of clinical treatment of the condition. Nevertheless, it was not possible to systematically investigate the MALAT1-ALKBH5 signaling axis in a mouse model or in patients’ samples, which would be our aims of further studies. Due to limited funding, the interactions between MALAT1 and ALKBH5, as well as the m6A methylation level in HPV-positive cervical cancer or clinical patient samples, were not evaluated in this study.

## Supplementary Material

Supplemental Material

## Data Availability

The data generated in the present study are included in the figures and/or tables of this article.
