# Supplementary material for: The involvement of MALAT1-ALKBH5 signaling axis into proliferation and metastasis of human papillomavirus-positive cervical cancer
Source: Cancer Biol Ther. 2023 Aug 28;24(1):2249174. doi: 10.1080/15384047.2023.2249174 (PMC10464551; doi:10.1080/15384047.2023.2249174)
Supplement: Supplemental Material [file KCBT_A_2249174_SM9107.zip › Supplemental figures/Figure Legends.docx]

**Supplementary Figures**

Fig. S1 Effect of FTO and METTL3 on MALAT1 expression in HPV-positive cervical cancer cells. CaSki and HeLa cells were transfected with siFTO or siMETTL3, respectively. After that, the mRNA expression level of FTO, METTL3 and MALAT1 were examined by RT-qPCR.

Fig. S2 Effect of MALAT1 on cell proliferation and metastasis in zebrafish xenograft model.

HeLa and CaSki cells transfected with NC or si-MALAT1 were labeled with DM-DiI and injected into zebrafish larvae yolk, respectively. After 96h, the zebrafish larvae were mounted using 1.2% low-melting gel, and the photographs were taken under stereomicroscope for quantification purpose. Representative images were shown.

Fig.S3 Overexpression of MALAT1 on ALKBH5 in HPV-positive cervical cancer cells.

HeLa and CaSki cells were transfected with empty vector or pcDNA3.1-MALAT1 plasmid. After that, the mRNA expression level of MALAT1, ALKBH5 was examined using RT-qPCR.

Fig. S4 Effect of ALKBH5 on cell proliferation and metastasis in zebrafish xenograft model.

HeLa and CaSki cells transfected with NC or si-ALKBH5 were labeled with DM-DiI and injected into zebrafish larvae yolk, respectively. After 96h, the zebrafish larvae were mounted using 1.2% low-melting gel, and the photographs were taken under stereomicroscope for quantification purpose. Representative images were shown.

Fig. S5 Overexpression of MALAT1 and ALKBH5in HPV-positive cervical cancer cells.

(A and B) HeLa and CaSki cells were transfected with empty vector or pcDNA3.1-MALAT1 plasmid. After that, the mRNA expression level of MMP2 and MMP9 was examined using RT-qPCR. (C and D) Empty vector or pcDNA3.1-ALKBH5 was transfected into HeLa and CaSki cells. The mRNA and protein level of ALKBH5 were analyzed by RT-qPCR and western blot using GAPDH as control. OE, overexpression.

Fig. S6 Effect of ALKBH5 on MMP2 and MMP9 expression.

(A and B) HeLa and CaSki cells were transfected with negative control and siALKBH5 (si1-ALKBH5 and si2-ALKBH5). The mRNA expression level of MMP2 and MMP9 was analyzed by RT-qPCR. (C and D) HeLa cells were transfected with negative control and siALKBH5. The half-life of MMP2 and MMP9 mRNA was analyzed using Actinomycin D Pulse-Chase Assay. (E) The expression of PVT1 was analyzed using RT-qPCR after transfection of negative control and siALKBH5 into HeLa cells. (F) The expression of PVT1, MMP2 and MMP9 was examined by RT-qPCR after knockdown of PVT1 in HeLa cells.
