# Supplementary figures and images for: The involvement of MALAT1-ALKBH5 signaling axis into proliferation and metastasis of human papillomavirus-positive cervical cancer
Source: Cancer Biol Ther. 2023 Aug 28;24(1):2249174. doi: 10.1080/15384047.2023.2249174 (PMC10464551; doi:10.1080/15384047.2023.2249174)

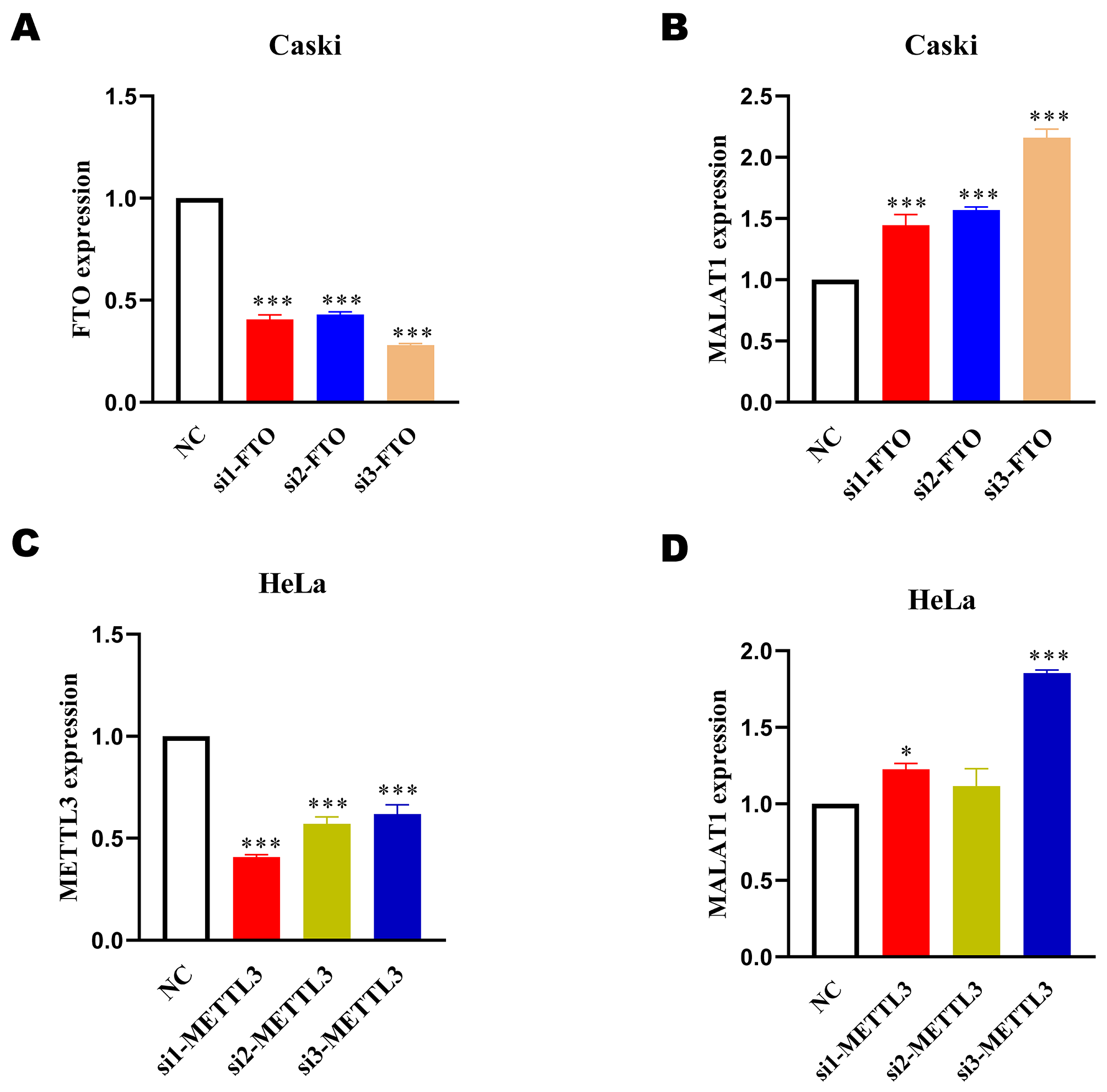

Supplement: Supplemental Material [file KCBT_A_2249174_SM9107.zip › Supplemental figures/Figure S1.tiff]

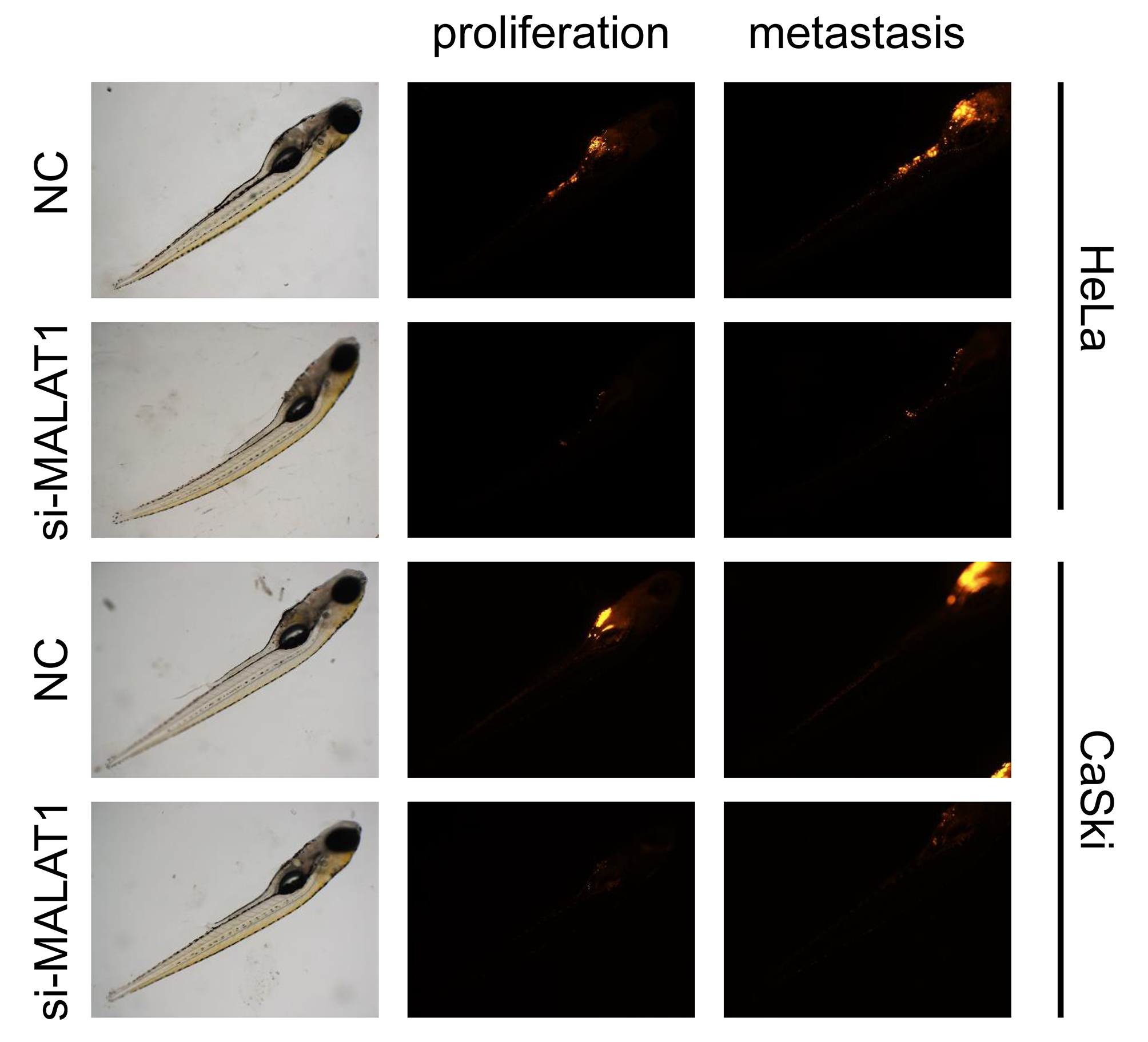

Supplement: Supplemental Material [file KCBT_A_2249174_SM9107.zip › Supplemental figures/Figure S2.tif]
